# Supplementary material for: Solvent-Free Synthesis of MgO-Modified Biochars for Phosphorus Removal from Wastewater
Source: Int J Environ Res Public Health. 2022 Jun 24;19(13):7770. doi: 10.3390/ijerph19137770 (PMC9265722; doi:10.3390/ijerph19137770)
Supplement: Supplementary file 1 [file ijerph-19-07770-s001.zip › ijerph-1764292-Supplementary.pdf]

Supplementary information

Solvent-free synthesis of MgO-modified biochars for phosphorus removal from wastewater

Siyu Xu[1#], De Li[1, 2#], Haixin Guo[1], Haodong Lu[3], Mo Qiu[1], Jirui Yang[1,*], Feng Shen[1,*]

[1] Agro-Environmental Protection Institute, Ministry of Agriculture and Rural Affairs, No. 31 Fukang Road, Nankai District, Tianjin 300191, China.

[2] College of Resources and Environment, Huazhong Agricultural University, No.1, Shizishan Street, Hongshan District, Wuhan 430070, China.

[3] Department of Chemical Engineering, University of Alberta, Edmonton, Alberta T6G 1H9, Canada

[#] Siyu Xu and De Li contributed equally to this manuscript.

**Corresponding author*: Dr. Jirui Yang, E-mail address: jiruiyang@163.com

**Corresponding author* (handling correspondence at all stages): Dr. Feng Shen,

E-mail address: shenfeng@caas.cn

The adsorption kinetic curves of phosphorus were analyzed with two kinetic models namely pseudo-first-order model and pseudo-second-order model, expressed by the following equations (1) and (2), respectively [46]:

pseudo-first-order model: $q_t = q_e(1 - e^{-k_1 t})$ (1)

pseudo-second-order model: $q_t = \frac{q_e(k_2 q_e^2 t)}{1 + k_2 q_e t}$ (2)

Where q_t and q_e (mg g^{-1}) are the adsorption capacity of phosphorus at time t and at equilibrium, respectively; k_1 (h^{-1}) and k_2 (g mg^{-1} h^{-1}) are the rate constants of the pseudo-first-order model and the pseudo-second-order model, respectively.

The data of adsorption isotherms were fitted with Langmuir and Freundlich models given by (3) and (4) [67]:

Langmuir model: $q_e = \frac{K_L q_{max} C_e}{1 + K_L C_e}$ (3)

Freundlich model: $q_e = K_F C_e^{\frac{1}{n}}$ (4)

Where q_e and q_{max} (mg g^{-1}) are the equilibrium phosphorus adsorption capacity and the maximum phosphorus adsorption capacity, respectively; K_L (L mg^{-1}) is the Langmuir constant; C_e (mg L^{-1}) is the equilibrium concentration; K_F ($mg^{(1-1/n)}$ $L^{1/n}$ g^{-1}) and n are Freundlich constants related to adsorption capacity and adsorption intensity, respectively.

The changes of Gibbs free energy ($\triangle G^0$ (kJ mol^{-1})), entropy ($\triangle S^0$ (J mol^{-1} K^{-1})) and enthalpy ($\triangle H^0$ (kJ mol^{-1})) were calculated by the following equations (5) and (6) [17,49]:

$\triangle G^0 = -RT \ln K_L$ (5)

$\triangle G^0 = \triangle H^0 - T\triangle S^0$ (6)

where R is the universal gas constant (8.314 J mol^{-1} K^{-1}); T is the absolute temperature (K); K_L is the Langmuir adsorption equilibrium constant.

Table S1. Elemental composition of MgO-modified biochars at different pyrolysis temperatures.

Adsorbent	Mg (%)	C (%)	H (%)	N (%)
2MgO/BC-250-0.5	12.72	37.44	4.38	0.27
2MgO/BC-450-0.5	27.62	29.94	2.20	0.26
2MgO/BC-650-0.5	28.89	27.75	1.32	0.25
2MgO/BC-850-0.5	31.16	26.71	0.61	0.23

Table S2. Thermodynamic parameters obtained from adsorption isotherms of phosphorus on 2MgO/BC-450-0.5 at 298-318 K.

T (K)	$\triangle G^0$ (kJ mol^{-1})	$\triangle S^0$ (J mol^{-1} K^{-1})	$\triangle H^0$ (kJ mol^{-1})
298	-16.5	103	14.2
308	-17.8		
318	-18.6		

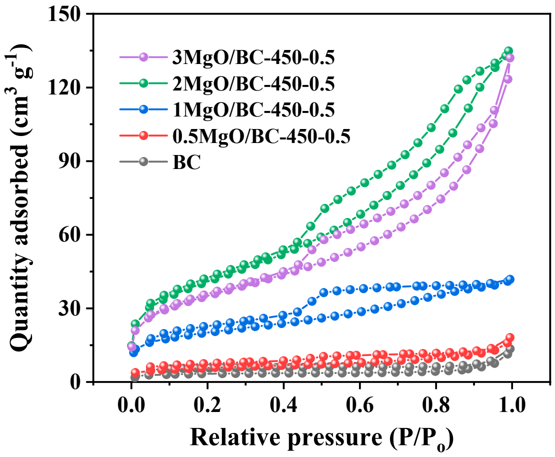

Figure S1. N_2 adsorption-desorption isotherms of prepared samples.

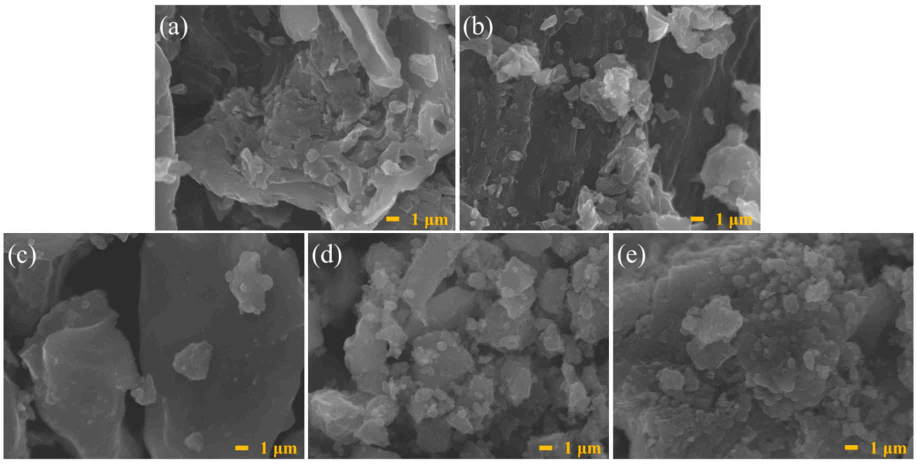

Figure S2. SEM images of (a) BC, (b) 0.5MgO/BC-450-0.5, (c) 1MgO/BC-450-0.5, (d) 2MgO/BC-450-0.5, (e) 3MgO/BC-450-0.5.

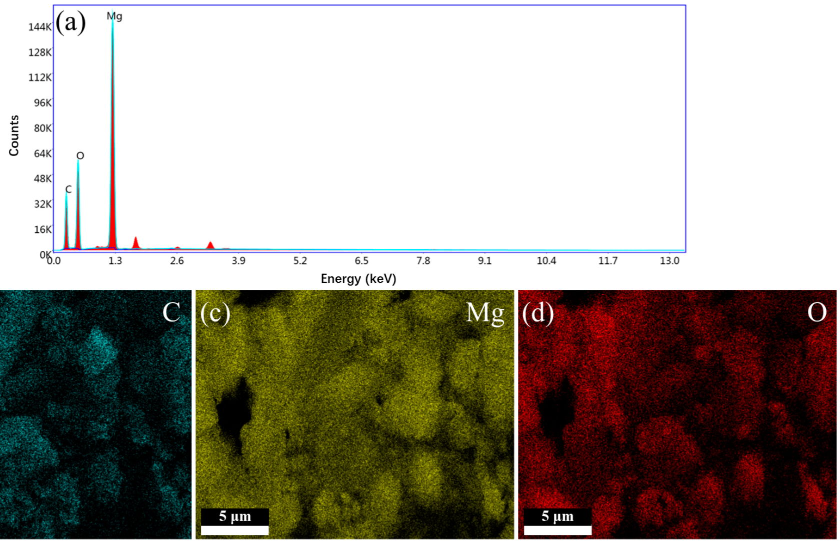

Figure S3. EDS spectrum pattern (a) and EDS mapping of (b) C, (c) Mg, (d) O of 2MgO/BC-450-0.5.

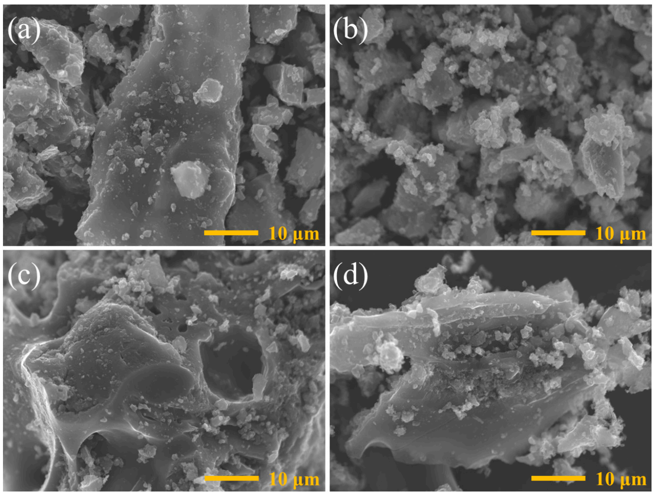

Figure S4. SEM images of MgO-modified biochars at different pyrolysis temperatures: (a) 2MgO/BC-250-0.5, (b) 2MgO/BC-450-0.5, (c) 2MgO/BC-650-0.5, and (d) 2MgO/BC-850-0.5.

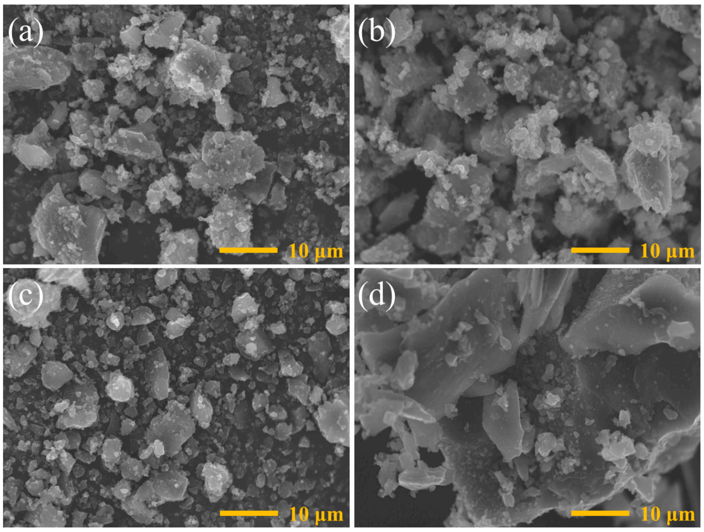

Figure S5. SEM images of MgO-modified biochars at different ball milling times: (a) 2MgO/BC-450-0.25, (b) 2MgO/BC-450-0.5, (c) 2MgO/BC-450-2, and (d) 2MgO/BC-450-10.

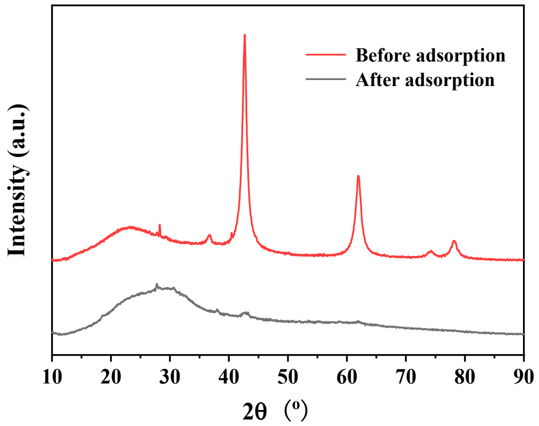

Figure S6. XRD patterns of 2MgO/BC-450-0.5 before and after adsorption.
